# Supplementary material for: The impact of wearing compression hosiery and the use of assistive products for donning and doffing: A descriptive qualitative study into user experiences
Source: PLoS One. 2024 Dec 26;19(12):e0316034. doi: 10.1371/journal.pone.0316034 (PMC11670924; doi:10.1371/journal.pone.0316034)
Supplement: S1 File — (PDF) [file pone.0316034.s001.pdf]

Interview guide qualitative study into user experiences wearing compression hosiery

Individual interviews with wearers of compression hosiery

NOTES. Before taking part in the study, the participant received an information letter and a consent form. Check that these have been signed/returned and that the participant has received a copy.

Introduction

Thank you for taking part in this interview.

The purpose of the interview is to identify your experiences with wearing therapeutic elastic stockings and putting on/removing them. What are bottlenecks, wishes and needs in this regard? If you agree, we will record this conversation with a voice recorder/recording in MS Teams for later typing. The data can then be analysed. All data will be stored and processed anonymously.

Is everything clear? Are you still willing to cooperate? If so, I will now switch on the voice recorder/recording.

Main question	Further questions (Examples)	Topic
I would like to take some personal information from you first.<br>How old are you?		Demographic data
How long have you been wearing CH?		
Why are you wearing CH?	What causes fluid retention in the legs?<br>Has any disease/diagnosis been established?	

What is your family and living situation?		
What is you educational background?	What is your highest educational level?	
Please describe the process by which the provision of CH was made.	- Which healthcare professionals were involved?<br>- Were you explained why wearing CH is necessary?<br>- How long did it take you to get the CH?<br>- Do you know when and for how long to wear the CH?<br>- Were you explained how to don/doff the CH?<br>- What was your experience of the provision process?<br>- What would you have liked to see different?<br>- What should be improved?	The CH provision process<br>- Role of healthcare professional<br>- Information about CH<br>- Self-reliance<br>- Impact CH<br>- Experiences from the provision process
How does wearing CH impact you?	- How do you feel about wearing CH?<br>- What are the advantages and disadvantages of wearing CH?<br>- Does your experience match what you thought about wearing stockings before?<br>- Was consideration given to the impact of wearing CH?<br>o If so:<br>- In what way?	Experiences of wearing CH<br>- Impact

	- What was your experience?<br>o If not, would you have liked to obtain information about it?	
How often and for how long do you actually wear the CH?	- Why do you wear/not/not always wear the CH?<br>- Do you appreciate wearing the stocking? Why yes/no?	Experiences of wearing CH<br>- Adherence
How does the process of donning and doffing CH proceed?	- Do you don the CH yourself?<br>o If yes:<br>- How do you do it?<br>- How much effort does it take?<br>- Do you use an assistive product to do so?<br>o If no:<br>- Why not?<br>- Have you tried an assistive product before?<br>- Who does don the CH?<br>- How do you feel about not being able to don the CH yourself?<br>- Would you like to be able to do it yourself or do you not mind having help?<br>- Does dependence on help hinder you in organising your day and	Experiences of donning and doffing CH and the use of APD<br>- Donning/doffing<br>- Self reliance<br>- Use of assistive products

	carrying out activities?<br>o If so:<br>- In what way?<br>- How do you experience this?<br>- Do you doff the CH yourself?<br>o If yes:<br>- How do you do it?<br>- How much effort does it take?<br>- Do you use an assistive product to do so?<br>o If no:<br>- Why not?<br>- Have you tried an assistive product before?<br>- Who doffs the CH?<br>- How do you feel about not being able to doff the CH yourself?<br>- Would you like to be able to do it yourself or do you not mind having help?<br>- Does dependence on help hinder you in organising your day and carrying out activities?<br>o If so:<br>- In what way?<br>How do you experience this?	

If an assistive product is used: How was the process of providing the assistive product?	- Which healthcare professionals were involved?<br>- Who initiated the use of a donning/doffing device?<br>- Were you explained how to use the donning/doffing device?<br>- Did you practise using the device with the healthcare professional?<br>o If yes: At home or at the healthcare professional's practice?<br>- Was the device reimbursed?<br>- How long did it take you to get the device?<br>- How did you experience the provision process of the donning/doffing device?<br>- Are you satisfied with the process?<br>- What would you have liked to see different?<br>- What should be improved?<br>- Do you have an idea of the cost of the device?<br>-Would you be able and willing to pay for the assistive product if it was	The provision process of the assistive product<br>- Role care professionals<br>- Proceeding provision process<br>- Information<br>- Training<br>- Self-reliance<br>- Experiences provision process

	not reimbursed by your health insurance?	
How does using a donning/doffing device impact you?	- How do you feel about having to rely on an assistive product for donning and doffing?<br>- What are the advantages and disadvantages of the assistive product ?<br>- Does your experience agree with what you thought before you started using the assistive product?<br>- What are the consequences (advantages and disadvantages) of using the APD and being independent now?<br>- Did you anticipate this before?<br>- What could reduce the barriers?	Experiences of donning and doffing CH and the use of an assistive product<br>- Impact of the use of the assistive device for donning/doffing CH
What considerations did you make before deciding whether or not to use a donning/doffing assistive product?	- What was the reason you wanted to be able to don/doff the CH yourself? Think about continuing activities, staying healthy, meeting needs (deciding when to get up and go to bed).<br>- Did you estimate beforehand how likely it was that you would be able to	The personal characteristics that influence acceptance of assistive products<br>- Considerations with decisions to use an assistive device for donning/doffing CH

	don and doff the stocking by yourself? And was this a consideration in your decision to proceed?<br>- Do you have alternatives to using the assistive product<br><br>OR<br><br>- What is the reason why you do not want to use an assistive product?<br>(Difficulty, no problems with dependency, cost of the assistive product , nice to have someone come every day)<br>- Did you estimate beforehand how likely it was that you would be able to don and doff the stocking yourself with the assistive product ? And was this a consideration for not starting?<br>- Do you have alternatives to using the assistive product APD?	
What role did your partner, (grand)children, family and friends play in your decision to use an assistive device to don/doff the CH?	- Have you been encouraged to try to become independent and to try an assistive product APD?<br>o If so:	Role social environment

	- By whom?<br>- How did you experience this?<br>o If not:<br>- Did anyone discourage you from doing so?<br>o If so:<br>- Do you know why?<br>- How did you experience this?	
What was the role of the compression therapist, GP, home care, physiotherapist, skin therapist or other health care providers (to use or don't use an assistive device to don/doff CH)?	- Have you been encouraged by them to try to achieve self-reliance and to try an assistive product ?<br>o If yes:<br>- By whom?<br>- Did they refer you to an occupational therapist or other healthcare provider?<br>- How did you experience this?<br>o If no:<br>- Have any people advised against it to you?<br>- And why?<br>- How did you experience this?	Role social environment
Did the situation in your home influence the choice to use an assistive product for donning/doffing and the selection of which device?	- Did the furnishing of dimensions of the room where you don/doff your CH influence the choice of the assistive product?	Role physical environment

How do you personally feel about using assistive product?	- Do you find it interesting?<br>- Do you see the need of assistive product ?<br>- Do you have a need for it?<br>- Do you think you have the skills to use an assistive product?<br>- Are you willing to make the effort to use an assistive product?<br>- Have you thought about the consequences of being able to don/doff CH independently using an assistive product?<br>- What are the consequences for you?	The personal characteristics that influence acceptance of assistive products<br>- Attitude towards assistive devices
Experiences with assistive product	- How do you feel about using an assistive product? What kind of assistive product do you use for donning/doffing?<br>- Are you satisfied with the assistive product ?<br>- How long have you been using the assistive product ?<br>- Do you always use it?<br>- Does the assistive product still work well?<br>- Has your problem with the assistive product been solved?	Experiences with assistive products

Are there any topics you missed? Or other points you think are relevant to this interview?		

Concluding

Thank you for your contribution to this study. I would like to send you a summary of this interview soon. Would you please proofread it, add to it or correct it if necessary? This will improve the quality of the research. If I do not receive a response to the report within 2 weeks, I will assume that you have no further comments and agree.
