# Supplementary material for: The impact of wearing compression hosiery and the use of assistive products for donning and doffing: A descriptive qualitative study into user experiences
Source: PLoS One. 2024 Dec 26;19(12):e0316034. doi: 10.1371/journal.pone.0316034 (PMC11670924; doi:10.1371/journal.pone.0316034)
Supplement: Attachment — Submitted filename: PONE-D-24-27618 WORD.docx [file pone.0316034.s002.docx]

PLOS ONE

The impact of wearing compression hosiery and the use of assistive products for donning and doffing: A descriptive qualitative study into user experiences

--Manuscript Draft--

| Manuscript Number: | PONE-D-24-27618 |
| --- | --- |
| Article Type: | Research Article |
| Full Title: | The impact of wearing compression hosiery and the use of assistive products for donning and doffing: A descriptive qualitative study into user experiences |
| Short Title: | User experiences of wearing compression hosiery and using assistive products for donning and doffing |
| Corresponding Author: | Edith Hagedoren - Meuwissen, MSc  Zuyd University of Applied Sciences: Zuyd Hogeschool Heerlen, Limburg NETHERLANDS, KINGDOM OF THE |
| Keywords: | compression hosiery; compression stockings; compression therapy; user experiences; patient perception; impact; quality of life; donning; doffing; assistive products for donning and doffing; non-adherence; non-compliance |
| Abstract: | This qualitative study aimed to describe users’ experiences and needs related to wearing, donning, and doffing compression hosiery, and the provision process of compression hosiery and associated assistive products for donning and doffing. Adults who have to wear compression hosiery participated in semi-structured interviews.  Existing frameworks about the provision process and acceptance of assistive technology guided the topic list. The interviews were analyzed using directed content analysis. After 19 interviews, data saturation was reached. Three main themes were revealed: (1) becoming and being a compression hosiery wearer (or not), (2) wearing compression hosiery, and (3) donning and doffing compression hosiery. In cases of acute conditions, the participants reported feeling overwhelmed when they were first prescribed compression hosiery. In contrast, those with long-term complaints felt relieved. Participants considered advantages and disadvantages and then decided whether to wear compression hosiery. Despite mentioned beneficial effects from wearing compression hosiery, participants had to cope with a range of discomforts, including pinching, straining, sagging, and heat. Additionally, participants had difficulties with the appearance, and often tried to hide the compression hosiery. They mentioned problems with donning and doffing, which can result in dependency of home or informal care, which stopped some participants from wearing. In general, participants were not aware of the full range of assistive products for donning and doffing, but were interested in them.  In conclusion, wearing compression hosiery has a large impact on a person’s life because of its lack of comfort, unattractive appearance, and possible loss of independence through the need of donning and doffing support. These are expressed reasons for non-adherence, in addition to a lack of understanding of the importance of wearing and the consequences of not wearing compression hosiery. Easy-to-find independent information and more attention to donning and doffing during the fitting appointment of compression hosiery are recommended. |
| Order of Authors: | Edith Hagedoren - Meuwissen, MA |
|  | Uta Roentgen, PhD |
|  | Sandra Zwakhalen, PhD |
|  | Loek van der Heide, PhD |
|  | Marie Josee van Rijn, PhD, MD |
|  | Ramon Daniels, PhD |
| Additional Information: |  |
| Question | Response |
| Financial Disclosure | Yes |

| Enter a financial disclosure statement that describes the sources of funding for the work included in this submission. Review the [submission guidelines](http://journals.plos.org/plosone/s/submission-guidelines#loc-financial-disclosure-statement) for detailed requirements. View published research articles from [PLOS ONE](https://journals.plos.org/plosone/) for specific examples.  This statement is required for submission and will appear in the published article if the submission is accepted. Please make sure it is accurate. |  |
| --- | --- |
| Funded studies  Enter a statement with the following details:   - Initials of the authors who received each award - Grant numbers awarded to each author - The full name of each funder - URL of each funder website - Did the sponsors or funders play any role in the study design, data collection and analysis, decision to publish, or preparation of the manuscript? |  |
| Did you receive funding for this work? |  |
| Please add funding details.  as follow-up to "Financial Disclosure  Enter a financial disclosure statement that describes the sources of funding for the work included in this submission. Review the [submission guidelines](http://journals.plos.org/plosone/s/submission-guidelines#loc-financial-disclosure-statement) for detailed requirements. View published research articles from [PLOS ONE](https://journals.plos.org/plosone/) for specific examples.  This statement is required for submission and will appear in the published article if the submission is accepted. Please make sure it is accurate. | This qualitative study was conducted in preparation of a cost-effectiveness study on an optimized provision process of assistive products for donning and doffing compression hosiery. The cost-effectiveness study is funded by ZonMw, project number 10310042110001. “ZonMw programs and funds research and innovation in health, healthcare and well-being, encourages the use of this knowledge and highlights knowledge needs.” [(https://www.zonmw.nl/en)](http://www.zonmw.nl/en)) The grant was awarded to the organizations involved, rather than to individual researchers. The funders had no role in study design, data collection and analysis, decision to publish, or preparation of the manuscript. |

| Funded studies  Enter a statement with the following details:   - Initials of the authors who received each award - Grant numbers awarded to each author - The full name of each funder - URL of each funder website - Did the sponsors or funders play any role in the study design, data collection and analysis, decision to publish, or preparation of the manuscript? |  |
| --- | --- |
| Did you receive funding for this work?" |  |
| Please select the country of your main research funder (please select carefully as in some cases this is used in fee calculation).  as follow-up to "Financial Disclosure  Enter a financial disclosure statement that describes the sources of funding for the work included in this submission. Review the [submission guidelines](http://journals.plos.org/plosone/s/submission-guidelines#loc-financial-disclosure-statement) for detailed requirements. View published research articles from [PLOS ONE](https://journals.plos.org/plosone/) for specific examples.  This statement is required for submission and will appear in the published article if the submission is accepted. Please make sure it is accurate. | NETHERLANDS - NL |
| Funded studies  Enter a statement with the following details:   - Initials of the authors who received each award - Grant numbers awarded to each author - The full name of each funder - URL of each funder website - Did the sponsors or funders play any role in the study design, data collection and analysis, decision to publish, or preparation of the manuscript? |  |
| Did you receive funding for this work?" |  |
| Competing Interests | The authors have declared that no competing interests exist. |

| Use the instructions below to enter a competing interest statement for this submission. On behalf of all authors, disclose any [competing interests](http://journals.plos.org/plosone/s/competing-interests) that could be perceived to bias this work—acknowledging all financial support and any other relevant financial or non- financial competing interests.  This statement is required for submission and will appear in the published article if the submission is accepted. Please make sure it is accurate and that any funding sources listed in your Funding Information later in the submission form are also declared in your Financial Disclosure statement.  View published research articles from  [PLOS ONE](http://journals.plos.org/plosone/) for specific examples. |  |
| --- | --- |
| NO authors have competing interests  Enter: The authors have declared that no competing interests exist.  Authors with competing interests  Enter competing interest details beginning with this statement:  I have read the journal's policy and the authors of this manuscript have the following competing interests: [insert competing interests here] |  |
| * typeset |  |
| Ethics Statement  Enter an ethics statement for this submission. This statement is required if the study involved:   - Human participants - Human specimens or tissue - Vertebrate animals or cephalopods - Vertebrate embryos or tissues | Written approval by the Medical Ethical Review Committee METC Z (METCZ20220025)  [(https://www.zuyderland.nl/zuyderland/bestuur-en-organisatie/metc-z/)](http://www.zuyderland.nl/zuyderland/bestuur-en-organisatie/metc-z/)) |

| - Field research   Write "N/A" if the submission does not require an ethics statement.  General guidance is provided below. Consult the [submission guidelines](http://journals.plos.org/plosone/s/submission-guidelines#loc-guidelines-for-specific-study-types) for detailed instructions. Make sure that all information entered here is included in the Methods section of the manuscript. |  |
| --- | --- |

| Format for specific study types  Human Subject Research (involving human participants and/or tissue)   - Give the name of the institutional review board or ethics committee that approved the study - Include the approval number and/or a statement indicating approval of this research - Indicate the form of consent obtained (written/oral) or the reason that consent was not obtained (e.g. the data were analyzed anonymously)   Animal Research (involving vertebrate animals, embryos or tissues)   - Provide the name of the Institutional Animal   Care and Use Committee (IACUC) or other relevant ethics board that reviewed the study protocol, and indicate whether they approved this research or granted a formal waiver of ethical approval   - Include an approval number if one was obtained - If the study involved non-human primates, add additional details about animal welfare and steps taken to ameliorate suffering - If anesthesia, euthanasia, or any kind of animal sacrifice is part of the study, include briefly which substances and/or methods were applied   Field Research  Include the following details if this study involves the collection of plant, animal, or other materials from a natural setting:   - Field permit number - Name of the institution or relevant body that granted permission |  |
| --- | --- |
|  |  |
| Data Availability  Authors are required to make all data underlying the findings described fully available, without restriction, and from the time of publication. PLOS allows rare exceptions to address legal and ethical concerns. See the [PLOS Data Policy](http://journals.plos.org/plosone/s/data-availability) and [FAQ](http://journals.plos.org/plosone/s/data-availability#loc-faqs-for-data-policy) for detailed information. | No - some restrictions will apply |

| A Data Availability Statement describing where the data can be found is required at submission. Your answers to this question constitute the Data Availability Statement and will be published in the article, if accepted. |  |
| --- | --- |
| Important: Stating ‘data available on request from the author’ is not sufficient. If your data are only available upon request, select ‘No’ for the first question and explain your exceptional situation in the text box. |  |
| Do the authors confirm that all data underlying the findings described in their manuscript are fully available without restriction? |  |
| Describe where the data may be found in full sentences. If you are copying our sample text, replace any instances of XXX with the appropriate details.   - If the data are held or will be held in a public repository, include URLs, accession numbers or DOIs. If this information will only be available after acceptance, indicate this by ticking the box below. For example: All XXX files are available from the XXX database (accession number(s) XXX, XXX.). - If the data are all contained within the manuscript and/or Supporting Information files, enter the following: All relevant data are within the manuscript and its Supporting Information files. - If neither of these applies but you are able to provide details of access elsewhere, with or without limitations, please do so. For example:   Data cannot be shared publicly because of [XXX]. Data are available from the XXX Institutional Data Access / Ethics Committee (contact via XXX) for researchers who meet the criteria for access to confidential data.  The data underlying the results presented in the study are available from (include the name of the third party | The consent form which is based on the template of the Dutch CCMO did not request permission to share anonymized data publicly. The data of participant who gave permission on the consent form to use their data for further research can be obtained form the first author upon request. It is also worth mentioning that the data are only available in Dutch. |

| and contact information or URL).   - This text is appropriate if the data are owned by a third party and authors do not have permission to share the data.   * typeset |  |
| --- | --- |
| Additional data availability information: |  |

Manuscript	[Click here to access/download;Manuscript;The impact of](https://www2.cloud.editorialmanager.com/pone/download.aspx?id=36830193&guid=297fcc2d-421d-4215-a93a-244016fc5784&scheme=1)[<https://www2.cloud.editorialmanager.com/pone/download.aspx?id=36830193&guid=297fcc2d-421d-4215-a93a-244016fc5784&scheme=1> wearing compression hosiery. DEF .docx](https://www2.cloud.editorialmanager.com/pone/download.aspx?id=36830193&guid=297fcc2d-421d-4215-a93a-244016fc5784&scheme=1)

1 **Full title**
2 **The impact of wearing compression hosiery and the use of assistive**
3 **products for donning and doffing: A descriptive qualitative study into user**
4 **experiences**

5

6 **Short title**
7 **User experiences of wearing compression hosiery and using assistive**
8 **products for donning and doffing**

9

10 **Authors**
11 Edith Hagedoren-Meuwissen^1,2^*, Uta Roentgen^1^, Sandra Zwakhalen^2,3^, Loek van der Heide^1^,
12 Marie Josee van Rijn^4^, and Ramon Daniels^1,2^

13

14 ^1^Research Centre on Assistive Technology in Care, Zuyd University of Applied Sciences,
15 Heerlen, the Netherlands
16 ^2^Department of Health Services Research, Maastricht University, Care and Public Health
17 Research Institute, Maastricht, the Netherlands
18 ^3^Academy of Nursing, Zuyd University of Applied Sciences, Heerlen, the Netherlands
19 ^4^Department of Vascular Surgery, Erasmus MC, Rotterdam, the Netherlands
20 * Corresponding author: [edith.hagedoren@zuyd.nl](mailto:edith.hagedoren@zuyd.nl) (EH)
21 Authors contributions
22 Edith Hagedoren-Meuwissen: conceptualization, funding acquisition, draft preparation,
23 methodology, formal analysis, investigation, writing - original draft
24 Uta Roentgen: supervision, conceptualization, funding acquisition, methodology, formal
25 analysis, writing - review & editing
26 Sandra Zwakhalen: supervision, conceptualization, methodology, writing - review & editing
27 Loek van der Heide: conceptualization, funding acquisition, writing - review & editing
28 Marie Josee van Tongerlo-van Rijn: writing - review & editing
29 Ramon Daniëls: supervision, conceptualization, funding acquisition, methodology, writing -
30 review & editing

31

32 Keywords:	compression	hosiery,	compression	stockings,	compression	therapy,	user
33 experiences, patient perception, impact, quality of life, donning, doffing, assistive products for
34 donning and doffing, non-adherence, non-compliance Are they MeSH terms? Are 6 keywords usually enough?

35

# 36 Abstract

37 This qualitative study aimed to describe users’ experiences and needs related to wearing,
38 donning, and doffing compression hosiery, and the provision process of compression hosiery
39 and associated assistive products for donning and doffing. Adults who have to wear
40 compression hosiery participated in semi-structured interviews. Existing frameworks about the
41 provision process and acceptance of assistive technology guided the topic list. The interviews
42 were analyzed using directed content analysis. After 19 interviews, data saturation was reached.
43 Three main themes were revealed: (1) becoming and being a compression hosiery wearer (or
44 not), (2) wearing compression hosiery, and (3) donning and doffing compression hosiery. In
45 cases of acute conditions, the participants reported feeling overwhelmed when they were first
46 prescribed compression hosiery. In contrast, those with long-term complaints felt relieved.
47 Participants considered advantages and disadvantages and then decided whether to wear
48 compression hosiery. Despite mentioned beneficial effects from wearing compression hosiery,
49 participants had to cope with a range of discomforts, including pinching, straining, sagging,
50 and heat. Additionally, participants had difficulties with the appearance, and often tried to hide
51 the compression hosiery. They mentioned problems with donning and doffing, which can result
52 in dependency of home or informal care, which stopped some participants from wearing. In
53 general, participants were not aware of the full range of assistive products for donning and
54 doffing, but were interested in them.
55 In conclusion, wearing compression hosiery has a large impact on a person’s life because of its
56 lack of comfort, unattractive appearance, and possible loss of independence through the need
57 of donning and doffing support. These are expressed reasons for non-adherence, in addition to
58 a lack of understanding of the importance of wearing and the consequences of not wearing
59 compression hosiery. Easy-to-find independent information and more attention to donning and
60 doffing during the fitting appointment of compression hosiery are recommended.

61

62	*Textboxes*

Textbox 1

Compression hosiery (CH) is manufactured in either a flat or circular knit by using elastic yarn. CH is available in standard sizes or can be custom made, both in various pressure classes (1–4) Please don’t speak classes, precise pressures and different lengths (below the knee, thigh length, and panty hose) [1].

Textbox 2

In the Netherlands, healthcare insurance reimburses two pairs of CH with a pressure class of 2–4 ( pressures) per year. A physician, mostly a general practitioner, dermatologist, vascular surgeon, or internist, makes the diagnosis. In most cases, the physician indicates what type of CH should be used. The provision of CH is often preceded by bandaging.(In what indications ?) Patients are free to choose the compression therapist (CT) who fits their CH. For non-ambulatory people, CH is fitted at home. In general, regular sizes or ready-to-wear CH are held in stock and patients receive them immediately; otherwise, CH has to be ordered with a maximum delivery time of 3 weeks. Some CTs first provide one pair of CH and arrange a check-up after 6 weeks. Others provide two pairs immediately and there is no further check-up.

63

Textbox 3

Assistive products for donning and doffing (APD^*^) CH provide support when donning and/or doffing CH is difficult, due to limitations in bending, reduced hand function, or CH with a high compression class (Pressures ?). They are available for CH wearers as well as (informal) caregivers. Some APD are only suitable for donning, others only for doffing, and some for both. There are more than 35 different APD that can be classified into four categories: (1) grip-enhancing aids, (2) resistance-reducing aids, (3) arm lengthening devices, and (4) electrical aids. (Photos?)

* A commonly used term for these devices has not been identified in the existing literature. For the purposes of this study, the acronym APD is used for both the singular and plural forms.

64

# 65 Introduction

66 Medical compression hosiery (CH) is a treatment option for venous and lymphatic diseases
67 such as varicose veins, chronic venous insufficiency, venous ulcers, lymphedema, lipedema (Ok in reference 2) , and acute
68 deep venous thrombosis [2]. The prevalence of CH wearers varies from one country to another.
69 Two percent of the Dutch adult population wears CH [3]. In 2023, CH was in the top 3 most
70 provided assistive products in the Netherlands that are reimbursed by healthcare insurance [3].
71 Despite the proven benefits and enhanced quality of life associated with wearing CH
72 [4], there remains a high non-adherence rate of 34% [5]. Non-adherence can exacerbate disease
73 progression and, consequently, lead to reduced mobility, pain, poor quality of life, and
74 increased healthcare costs [6]. Users’ experiences of wearing CH have been reported in several
75 studies, mainly with a focus on non-adherence. They describe various reasons for non-
76 adherence including a lack of knowledge, discomfort (pain, heat, and skin irritation), and
77 problems with the appearance of CH [6-10]. Moreover, donning and doffing CH are
78 burdensome and tedious tasks. Twenty-seven percent of CH wearers are not able to don CH
79 independently [7]. This inability leads to non-adherence or dependence on informal carers or
80 home care and, consequently, additional healthcare costs due to complications. About one third
81 of Dutch CH wearers receive home care for donning and doffing. Of note, in the Netherlands
82 home care is already stretched by understaffing. Assistive products for donning and doffing
83 CH (APD*) are available to simplify donning and doffing. With an APD, 93% of users can
84 successfully don their CH [7]. Nevertheless, only 15% of Dutch CH wearers receive an APD
85 from their health insurance [3].

* A commonly used term for these devices has not been identified in the existing literature. For the purposes of this study, the term “APD” is used for both the singular and plural forms.

86 The available studies on CH provide a relatively superficial description of the
87 experiences of CH wearers and do not provide insight into their experiences using APD. A
88 deeper understanding of the experiences and needs of CH wearers can be beneficial to optimize
89 the provision of information about CH and APD to enhance adherence and independence. Thus,
90 we aimed to describe users’ experiences and needs related to obtaining, wearing, donning, and
91 doffing CH and obtaining and using APD.

92

# 93 Materials and methods

94 The research paradigm social constructivism is applied to understand and describe human nature with its multiple subjective realities and truths, constructed by and between individuals (not clear..)
95 [11]. This descriptive, qualitative study used the lens of CH wearers, focusing on their feelings,
96 perceptions, and experiences [11]. Individual semi-structured interviews were conducted to
97 obtain an in-depth understanding of wearing CH and using APD.

99

## 100 Study population

101 The study population consisted of compliant adult wearers of CH with a compression class of
102 II, III or IV (Please precise pressures )who had been wearing CH for between 6 months and 3 years, so that each person
103 had sufficient experience and could remember the provisioning process. People were excluded
104 if they were living in a nursing home, had severe cognitive impairments, or did not speak
105 Dutch. Purposive sampling was used to maximize variations in experiences and impacts [12]
106 as well as gender and the type of CH. As a result, more non-compliant CH wearers were
107 interviewed.

108

## 109 Interview guide

110 The topic list was guided by frameworks [13] about the provision process [14] and assistive
111 technology acceptance [15]. The interview guide comprised six main topics: (1) the CH
112 provision process, (2) the experiences of wearing CH, (3) the experiences of donning and
113 doffing CH and the use of APD, (4) the provision process of APD, (5) the personal
114 characteristics that influence acceptance of assistive products, and (6) the role of the social and
115 physical environment. The Nictiz framework [14] for the provision of assistive technology
116 provided the subtopics regarding the CH and APD provision process. Subtopics in personal
117 characteristics and the role of social and physical environment emerged from the model of
118 assistive technology acceptance [15]. Each participant provided the following demographic
119 information: age, gender, family situation, education level, diagnosis, comorbidities, type of
120 CH, wearing time, adherence, use of an APD, and level of independence in donning and
121 doffing.

122

## 123 Procedure

124 After collecting the demographic information, the interview first focused on the CH provision
125 process and the attention that was paid to donning and doffing during the fitting and delivery
126 of CH. It then zoomed in on the personal experiences of wearing CH, donning and doffing, and
127 using an APD. Lastly, influencing personal characteristics, as well as the role played by the
128 social and physical environment, were examined. All interviews were conducted by the same
129 researcher (EH) who has a Master of Arts degree in change management; she has been trained
130 as an occupational therapist but has mainly been working as a researcher in recent years. She
131 was involved in previous research on donning and doffing CH by caregivers and about usability
132 of APD. A pilot interview revealed that adaptation of the interview guide was not necessary.
133 The data from the pilot interview was so rich and valuable that it was incorporated into the
134 final results. After approval by the Medical Ethical Review Committee METC Z
135 (METCZ20220025), the participants were recruited from 16-5-2022 until 30-09-2022 via
136 patient associations, compression therapists (CTs), home care organizations, and social media.
137 Those who met the inclusion criteria received an information letter about the study. After
138 agreeing to participate, they signed a written informed consent form. Inclusion was continued
139 until saturation was reached. Each interview was conducted face-to-face at the participant’s
140 home or via video call, depending on the participant’s residence, digital skills, and preference.

141

## 142 Data analysis

143 With the participant’s permission, each interview was audio recorded and transcribed verbatim.
144 Member checking was utilized: A summary of the interview was sent to each participant within
145 1 month of the interview to check for agreement. If there was no response within 2 weeks, then
146 agreement was assumed. Interview data were analyzed using directed content analysis, guided
147 by the topics of the interview guide. Coding was conducted by two researchers (EH and UR)
148 using Atlas.ti ([www.atlasti.com](http://www.atlasti.com/)). First, the interviews were read and re-read to get an
149 overarching view of the content and to get an initial view of the themes and non-preconceived
150 topics. EH and UR started by coding two interviews together to calibrate the process. All of
151 the data fit into the coding scheme. After coding all of the interviews, EH and UR ordered
152 similar categories into broader categories and then grouped them into major themes with
153 subthemes. Finally, EH and UR described the key findings by theme [16–18].

154

## 155 Quality criteria

156 The following quality criteria described by Frambach et al. [12] were considered.
157 *Credibility*: The semi-structured interviews were conducted using a predefined topic
158 list, based on a framework for the provision of assistive products. Each participant was asked
159 to provide feedback on the transcript and summary of the interview (member checking).
160 Investigator triangulation was applied as two researchers coded the data.
161 *Transferability*: Experiences were described in detail and in their context when it
162 mattered to reach thick descriptions and to make the findings meaningful. In the Results,
163 participants are identified by numbers to allow the reader to assess the context. The sampling
164 strategy strived for maximum variation concerning experiences and impact. In the Discussion,
165 the findings are compared with the existing literature from different settings.
166 *Dependability*: Data were collected until no new information emerged (saturation).
167 During the interview period, the researcher (EH) was flexible and open to topics and the process
168 (e.g., the inclusion criteria were broadened). By continuously reexamining the data using
169 insights that emerged during analysis (iterative data analysis), it became clear that adding
170 participants who were non-compliant with wearing CH could provide additional interesting
171 data.
172 *Confirmability*: The interviewer (EH) herself wears CH. She could see the reality
173 through the same “lens” as the participants and thanks to her experience and knowledge, she
174 could ask probing questions to obtain more in-depth information. She reflected critically on
175 her role throughout the research process (reflexivity). Based on the audio recording of the pilot
176 interview, the interviewer (EH) received feedback on her interview technique from a colleague
177 researcher (UR), paying special attention to the neutrality of the questioning (peer debriefing).
178 Steps and decisions taken in the research and their motives were documented to provide an
179 audit trail.

180

# 181 Results

182 Each interview lasted for 30–45 minutes. After 19 interviews, data saturation was reached and
183 no new insights emerged. In response to member checking, one summary was modified: The
184 participant felt the summary did not express his experiences appropriately. The summary
185 indicated that the participant did not feel up to learn to don and doff CH himself because of
186 frustration and stress. However, the participant replied that this was not possible because of
187 coordination problems and loss of strength. He added that there was nothing to be ashamed of
188 in asking for help.

189

| **Themes** | **Subthemes** |
| --- | --- |
| **Becoming and being a CH wearer (or not)** | Feeling overwhelmed or relieved |
|  | Feeling not well-informed |
|  | Weighing the pros and cons |
|  | Feeling supported by relatives and indifferent with  reactions to others |
|  | Handling CH with care |
| **Wearing CH** | Experiencing relief and functional gain |
|  | Constant dealing with discomfort |
|  | Avoiding CH attracts attention |
| **Donning and doffing CH** | Learning to cope with difficulties |
|  | Finding the right place in different circumstances |
|  | Appreciating independence |
|  | Willingness to use an APD depends on the need and  perceived benefits |
|  | Being unaware of the range of APD |
|  | Satisfied about the use of APD |

190

## 191 Participants

192 Table 1 provides detailed information on the 19 participants. The participants wore CH because
193 of various conditions: lymphedema, lipalgia, chronic venous insufficiency, varicose veins, and deep
194 venous thrombosis. One participant could not explain why he wore CH. The participants’
195 backgrounds also varied in terms of the education level and living situation. Fourteen
196 participants wore CH (nearly) every day and four participants wore CH occasionally or never.
197 Thirteen participants could don and/or doff CH themselves, five received help from informal
198 carers, and three received home care. Of those three participants, two were able to don CH by
199 themselves, but they needed help to doff CH. Thirteen participants used an APD.

## 200 Table 1. Characteristics of the participants.

|  | Age (years) | Gender | Years of wearing CH | Type of CH | Compression class | Who is donning/ doffing? | Use of an APD | Family situation | Education level | Diagnosis | Other diseases/ disabilities | Adherence |
| --- | --- | --- | --- | --- | --- | --- | --- | --- | --- | --- | --- | --- |
| P1 | 57 | Female | 3 | Thigh, circular knit, made- to-measure, open toe | Left: 3 Right:  2 | Self | Silk sock and gloves | Married | Bachelor | Secondary lymphedema | Arm fracture (healed) | + |
| P2 | 29 | Female | 1.5 | Pantyhose, flat knit, made-  to-measure | 2 | Self | Only gloves | Single | Bachelor | Lipalgia | - | + |
| P3 | 21 | Female | 1 | Knee only left leg, flat knit, ready to wear, open toe | 2 | Self | Foot slip | Living with parents | Bachelor | Deep venous thrombosis | - | + |
| P4 | 80 | Male | 2.5 | Thigh, flat knit, made-to-  measure, open toe | 3 | Self | Foot slip | Living together | Master | Secondary lymphedema | Prostate cancer (cured) | + |
| P5 | 48 | Trans man | 4 | Knee, flat knit, made-to-  measure, open toe | 2 | Informal care | Only gloves | Single | Vocational  college | Lipalgia and  lymphedema | Transient ischemic attack (two  times), obesity, prolapses | + |
| P6 | 87 | Female | 2 | Knee, flat knit, flat knit, ready to wear, open toe | 2 | Donning: self Doffing: informal care | Foot slip | Widow | Primary education | Edema | Arthrosis | + |
| P7 | 35 | Female | 1 | Knee, made-to-measure and  ready to wear, closed toe | 3 | Self (laborious) | - | Long-distance  relationship | Master | Primary lymphedema | - | + |
| P8 | 83 | Male | 2 | Knee, circular knit, ready to wear, open toe | 2 | Home care | Foot slip or Doff N’  Donner | Widow | Vocational college | Participant did not know | Partial spinal cord injury | + |
| P9 | 69 | Female | 3 | Knee and thigh, circular knit, ready to wear, open toe | 2 | Home care | Foot slip | Married | Primary education | Deep venous thrombosis | Crushed arm | + |
| P10 | 81 | Female | 4 | Knee, circular knit, ready to  wear, open toe | 2 | Home care | Silk sock | Widow | Primary  education | Edema | Loss of strength, dizziness | + |
| P11 | 63 | Male | 2 | Knee and thigh, flat knit,  made-to-measure | 4 | Self | Silk sock | Married | Vocational  college | Secondary lymphedema | Prostate cancer (cured) | + |
| P12 | 69 | Female | 1 | Knee, circular knit, ready to wear | 2 | Informal care | Doff N’ Donner | Living together | Vocational college | Edema | Arthrosis, breast cancer | + |
| P13 | 78 | Male | 3 | Knee, circular knit, ready to  wear, open toe | 2 | Informal care | Foot slip | Married | Vocational  college | Venous insufficiency | Arthrosis in hip | + |
| P14 | 61 | Female | 5/6 | Pantyhose, flat knit, made-  to-measure, closed toe | 3 | Donning: self (laborious)  Doffing: informal care | Only gloves | Married | Bachelor | Primary lymphedema | - | - |
| P15 | 50 | Female | 6 | Knee, circular knit, ready to wear, open toe | 2 | Self | Foot slip | Married | Master | Deep venous thrombosis | - | + |
| P16 | 53 | Female | 6 | Thigh, circular knit, ready to  wear, open toe | 2 | Self | - | Married | Bachelor | Varicose veins | - | - |
| P17 | 71 | Female | >50 | Knee, circular knit, made-to- measure, open toe | 2 | Self (laborious) | Foot slip | Single | Primary education | Varicose veins, edema | Cardiac failure, diabetes, chronic obstructive pulmonary disorder, tendonitis,  fibromyalgia, obesity | +/- |
| P18 | 50 | Female | 24 | Thigh, circular knit, ready to  wear, open toe | 2 | Self | - | Married | Bachelor | Varicose veins | - | - |
| P19 | 59 | Female | 25 | Knee, circular knit, ready to  wear, closed toe | 2 | Self (laborious) | - | Married | Vocational  college | Varicose veins | Diabetes, Lyme disease,  fibromyalgia, back pain | - |

201

12

## 202 Becoming and being a CH wearer (or not)

203 Feeling overwhelmed or relieved
204 The participants had different reactions regarding the medical advice for wearing CH. Certain
205 participants, such as those dealing with long-standing conditions like lymphoedema or
206 lipoedema and who frequently received a late diagnosis, generally said they took a positive
207 attitude, expecting that CH would bring relief to their ailment (P7 and P11). The participants
208 with acute conditions (P3, P9, and P15), such as deep venous thrombosis (DVT), reported
209 feeling overwhelmed by the news that they would have to wear CH.
210 Before obtaining CH, the participants expressed that they could not imaging the impact
211 of wearing it (P8 and P15). During the CH fitting process, they felt that the impact of wearing
212 CH was often overlooked by both physicians and CTs (P3, P6, P7, P15, and P19). Even when
213 attention was given, the participants said it often had little impact due to the overwhelming
214 amount of new information they were provided. Some participants missed the attention given
215 to the impact of wearing CH; others did not. In addition, CH provision frequently came after
216 bandaging, which had an even larger impact than wearing CH. Hence, the impact of wearing
217 CH was initially perceived to be relatively low to the participants. They indicated that there
218 was a greater impact when they ultimately realized that wearing CH is usually a lifelong
219 commitment.
220 Many of the participants minded having to wear CH (P1, P2, P3, P7, P12, P14–P16,
221 P18, and P19). Others had little or no problem with wearing CH and were not ashamed of it
222 (P4, P6, P8, P11, P13, and P17)—remarkably, all men and women over 70 years of age. They
223 felt wearing CH is appropriate for their age (P6 and P17).
224 P1: *Accepting that you have lymphedema, if you don’t have it from birth, and wearing*
225 *CH becomes much more manageable if you have the right guidance and support. With*
226 *a dermatologist or skin therapist who listens to you and thinks with you.*
227 P1: *That has to do with my ability to put things into perspective and also when your*
228 *husband goes blind and you get cancer, how bad is this? You can’t compare and I also*
229 *sometimes think of myself as the most pathetic person in the whole world, but that is*
230 *the way things are and you don your CH and you go on.*

231

232 Feeling not well-informed
233 In many cases, the participants felt that they had not been fully informed by physicians,
234 especially regarding the aesthetic aspects of CH (P7 and P15). During CH fitting by their CTs,
235 the participants received extensive information, but they did not remember everything. Short
236 appointments hindered questions and discussions, resulting in unresolved queries during CH
237 use (P9 and P17). The participants explained that during the fitting appointment, they felt
238 confronted with a lot of new experiences (P10 and P17) and felt they had missed a lot of the
239 verbal information they had received. A few felt well informed (P1, P9, and P11); they had
240 stayed in a specialized treatment center for some weeks and had received all the required
241 information. The participants stressed that good advice and support from healthcare
242 professionals helps with the acceptance process (P1, P2, P4, P5, P9, and P11). Sometimes, CH
243 was fitted by a skin therapist (ST) (esthetician ?) instead of a CT. A few participants (P2, P4, and P5) indicated
244 that they appreciated the approach of the ST who was knowledgeable, took time for explanation
245 and emotions, answered questions, treated lymphedema ( ??? physiotherapist???), and paid attention to donning and
246 doffing, including fitting an APD.
247 Most of the participants were unaware of the specific type of CH they were wearing.
248 Almost all of the participants believed they were wearing made-to-measure CH, but only 8 of
249 the 19 participants were actually wearing made-to-measure CH. The majority could point out
250 why they must wear CH and the implications of not doing so (P1–P5, P7, P9, P12, P14, P16,
251 P18, and P19), but three participants (P6, P8, and P13) expressed a lack of awareness of the
252 reasons and significance behind wearing CH. Two participants (P6 and P10) did not know that
253 their health insurance covers the cost of two pairs of CH each year, leading them to wear the
254 same CH for multiple years.
255 Most of the participants commented on the limited freedom of choice and shared
256 decision-making regarding CH selection. For example, the choices in CH were primarily
257 restricted to color (black or skin tone), toe type, and, occasionally, length (P1, P3, P6, P8, P12,
258 and P14–P18). Only two participants (P4 and P11) mentioned that factors such as activities and
259 personal circumstances were considered, and they appreciated the fact that decisions were
260 reached through consensus. The participants emphasized the importance of evaluation after a
261 few weeks.
262 According to the participants, practical information is needed regarding wearing CH,
263 fit (tightness/looseness), wearing duration, suitability in warm weather, maintenance
264 (washing/drying), and CH lifespan (P3, P6, P7, P10, P15, and P17). The participants often
265 found information leaflets about CH to be unreadable due to small text and multiple languages
266 (P3 and P5). Some participants sought insights from other CH wearers and information on
267 recent advancements in compression therapy. The participants who searched the Internet found
268 fragmented and sometimes conflicting information, primarily on commercial websites (P1, P3,
269 P5, P14, and P15). The independent information sources mentioned by the participants
270 comprised websites of patient associations of DVT and lymphedema/lipalgia, and a specialized
271 treatment center for lymphedema. Additionally, some mentioned the patient associations on
272 social media. The participants emphasized the significance of critical evaluation when reading
273 information, as it may not always be impartial or reliable. P3 thought that tight trousers enhance
274 the effectiveness of CH and that CH color influences its effectiveness.
275 P3: *I found it [information] on the Thrombosis Foundation website […]. Other than*
276 *that, I’ve gone from website to website. I went over English and American websites, as*
277 *well as a couple of Belgian websites and a French one. Then you just read through that*
278 *and then you look at which things generally come up multiple times. So, I would have*
279 *liked it if there was just one website.*
280 P5: *With the stocking, you get such a thick booklet and then I still don’t know what to*
281 *do with it. [...] It’s not very informative. [...] A compact booklet would have been good*
282 *enough. And then in 20 languages and small print, I’m not going to bother with that.*

283

284 Weighing the pros and cons
285 Most of the participants perceived CH as a necessary evil (P1–P13, P15). They consistently
286 wore CH on a daily and continuous basis due to the perceived positive effects or concerns about
287 potential risks associated with discontinuation. However, the participants who reported
288 experiencing no immediate benefits from CH (P14, P16, P18, and P19) tended to wear them
289 inconsistently, despite the associated risks of wounds or infections. One participant (P17)
290 believed that there was no point in wearing CH occasionally, so she did not wear CH at all.
291 Two participants (P14 and P19) selectively wore CH when suffering or as a form of prevention,
292 for example, during extended travel. The reasons the participants mentioned they were not
293 willing to wear CH included pain or discomfort (P14, P16, P18, and P19), the appearance of
294 the CH (P16), and problems with donning and doffing (P14 and P17). On warm days, some
295 participants explained that when wearing CH, they experienced more symptoms of the disease
296 (P17), whereas others abstained from wearing CH because the heat makes it uncomfortable
297 (P16 and P19). P1, P7, and P14 augmented CH with a pneumatic compression device. Due to
298 discomfort, some participants explored alternatives such as padding bandages (P14), over-the-
299 counter compression class 1 (please precise pressure) CH (P18 and P19), or tightly fitted skinny jeans (P14). Some
300 participants expressed that the effort required for donning or doffing CH prevents them from
301 wearing it daily (P2, P17, and P19). CH that is easier to don (e.g., with a zipper) would increase
302 willingness to wear it (P17). P5, P14, P15, and P17 struggled with improperly fitting CH: It
303 was too long or too tight, sometimes leading to inconsistent usage.
304 P11: *If I didn’t have to wear them, I wouldn’t wear them. Nobody does it for fun. It’s*
305 *the same with hearing aids or glasses, if you don’t need them, you don’t put them on.*
306 P19: *It’s not that I don’t want to wear them, is it? It’s just that there’s so much stopping*
307 *me from wearing them.*
308 P18 [woman with varicose veins and occasional phlebitis]: *I think the discomfort of*
309 *wearing outweighs the occasional symptoms.*

310

311 Feeling supported by relatives and indifferent to reactions of others
312 The participants clearly stated that they decide whether or not to wear CH. The role of relatives
313 was mentioned by several participants (P2, P4, P5, P10, P11, P12, P14, P15, P16, and P18).
314 The participants stated that family members sometimes encouraged wearing CH (P17 and P19)
315 or were understanding and willing to support the participant when necessary—for example, in
316 donning (P1, P5, P6, P12, P13, P14, and P15). Some relatives helped to find solutions to
317 problems regarding CH. For example, P4’s partner modified his CH so that it no longer sagged.
318 Another repaired CH with a hole (P9).
319 The participants mentioned that negative reactions most often come from people who
320 are less close to them, for example, people telling them how horrible it must be to wear CH
321 (P11). Most of the participants said that these reactions have had no impact as they wear CH
322 because it is necessary, regardless of others’ opinions. They usually tended not to invest any
323 energy in trying to change people’s minds. The participants explained that they usually do not
324 talk to others about wearing CH, nor are they looking for peer contact to share experiences (P1,
325 P2, P4, P7, P9, P13, P14, P15, and P17). A few were members of a patient association or
326 followed its social media (P1 and P3).
327 P14: *My husband says: “You are old and wise enough to feel for yourself what you do*
328 *and what is possible.”*
329 P17: *My son frequently comments about not wearing the CH regularly at the beginning.*
330 *“Mom, that’s not good.” And my daughter-in-law honestly says, “Mom, it’s not wise.*
331 *You should wear them, but I understand why you aren’t wearing them.”*
332 P11: *I also speak to people who say, “I don’t understand why you wear those stockings*
333 *in summer then the leg [is] a bit thicker.” Then I say, “That’s because you don’t know*
334 *what you’re talking about because you don’t have this disease. Otherwise you would*
335 *be wearing them.”*

336

337 Handling CH with care
338 The participants said they handle their CH with care: They wash them separately (P10) and
339 keep their fingernails short or use anti-slip gloves, as long nails can damage CH (P1).
340 Nevertheless, some of the participants (P1, P6) reported that the stretch of the CH decreases
341 over time, and CH can wear out and become perforated (P2, P7, P9, and P10). P3 (incorrectly)
342 thought that machine washing and spinning should be avoided to preserve CH quality. Two
343 participants (P7 and P9) were satisfied about the warranty. In the Netherlands, health insurance
344 reimburses two pairs of CH per year. Two participants (P2 and P12) felt that this was not
345 enough because of wear and tear and because they had to rotate CH for washing. The
346 participants found CH to be expensive, keeping them from buying an extra pair (P5). Thus,
347 despite reduced pressure, CH are worn longer than 1 year so that they have more CH to rotate
348 and have more time for washing and drying.
349 *P2: That’s just wear and tear, so two CH in a year is not enough. I have two with a*
350 *hole in them now, you wear them out pretty fast because you have to wear and wash*
351 *them daily.*

## 352 Wearing CH

353 Experiencing relief and functional gain
354 The participants described that they experienced pressure from CH around their legs. While
355 some found the support beneficial (P6 and P15), others reported it to be unpleasant (P7, P12,
356 P14, P16, P17, and P18). Most participants reported positive effects associated with wearing
357 CH: ankles/legs there were not as thick (P1, P6, P7, P11, P12, and P19), no wounds (P12),
358 fewer varicose veins, no more pain in calves (P2), and enhanced standing and walking (P2).
359 Notably, the participants reported that when CH is not worn, leg swelling occurs (P1 and P7),
360 and some participants experienced discomfort, like problems with walking the following day
361 (P5) or a persistent sense of fatigue (P19). Those who observed positive effects generally
362 considered the benefits to outweigh the drawbacks. However, over time, CH elasticity
363 diminished, and symptoms reappeared, prompting some participants to desire more frequent
364 replacement (i.e., more than once a year) (P5 and P12).
365 P2: *But I did notice very clearly that when I was wearing them (CH) I could stand*
366 *longer at a bar table at a party or walk better than when I wasn’t wearing them. By the*
367 *end of the evening, I’m walking like a penguin. I was waddling, and when I’m wearing*
368 *these CH I can hold on much longer.*
369 P5: *If I don’t wear them, I can hardly walk the next day.*
370 P6: *I like the fit around the legs and the support around the legs. I have never taken*
371 *them off. That’s how comfortable I find them.*

372

373 Constant dealing with discomfort
374 Wearing CH could cause discomfort, pinching, and even pain according to many participants
375 (P2, P4, P8, P14, P15, and P18), which could also interfere with their activities (P1). A few
376 participants said they could not tolerate wearing CH all day for this reason (P14 and P19). The
377 participants stated that warm weather exacerbated the discomfort, as it tended to make them
378 feel more bothered than during the winter months (P1, P3, P4, P6, P9, P10, P11, P13, P16, P18,
379 and P19). According to some participants, the brand (P13) and length of CH seemed to have
380 an impact on comfort: All wearers of thigh-high CH complained about discomfort. The knitting
381 method also impacted comfort: P2 and P7 tried both and rated the comfort of the flat knit
382 higher. The label inside the CH can cause irritation (P10), but removal will void the warranty
383 (P5). P9 indicated that a skirt or thin trousers can creep up along CH. The majority of wearers
384 of thigh-high CH reported that this type tends to sag (P1, P4, P11, P12, P16, and P18), which
385 could be especially problematic when outdoors, as it requires pulling trousers down or lifting
386 skirts to put CH on properly again (P1 and P4). To prevent sagging, the participants sometimes
387 used skin glue (P11) or opted for tights or knee-high stockings rather thigh-high stockings. P4
388 sought creative solutions to improve the comfort of CH and to prevent sagging; however, tights
389 could make standing urination impossible for men. P2 mentioned that tights also could cause
390 uncomfortable pressure on the abdomen for women, particularly during menstruation, leading
391 to avoid wearing it. Additionally, the participants indicated that tights were even warmer than
392 CH and considered them to be too hot in the summer.
393 P19: *In the summer it bothers me. When it’s so hot. I can’t stand the heat very well*
394 *anyway. So, when I hear that it’s going to be a hot day, I don’t put them on.*
395 P17: *In the summer, when it’s so hot, I wear the CH every day. But [...] when it’s a little*
396 *less hot […] I’m not wearing them. I have a lot of trouble donning them.*

397

398 Avoiding CH attracts attention
399 When the participants discussed the impact of wearing CH, their focus frequently revolved
400 around the visual aspect of it (P6 and P16), and the implications for their clothing and footwear
401 choices (P1, P2, P3, P5, P7, P9, P10, P12, P15, P16, P18, and P19). The participants found
402 their CH to be ugly (P16, P18, and P19) and associated CH with old people (P3 and P5). Many
403 participants expressed issues with the color and material of their CH (P2, P14, and P15). They
404 stated that the color was not a suitable match for their skin tone (P2) and faded with washing.
405 According to several participants, there was little innovation in CH: The look of CH has been
406 the same for decades (P2 and P14). Some suggestions for improvement included using a silkier
407 material, adding glitter to the seam, or incorporating prints that resemble tattoos (P14 and P15).
408 The participants were often unaware that CH are available in hues other than skin tone and
409 black. Other participants felt conflicted about CH colors. They desired more variety to match
410 their clothing but often compromised for neutral colors due to the annual restriction of two
411 reimbursed pairs. P7 opted for made-to-measure CH because of the broader color selection.
412 Some participants said they drew attention away from CH by wearing conspicuous shoes or by
413 wearing colorful CH that are not recognized as such (P3 and P5); others would hide CH under
414 long trousers or dresses (P1, P2, P3, P9, and P10). P1 even bought a new wardrobe to hide her
415 CH. Remarkably, the men interviewed did not report issues about the appearance of CH at all.
416 Some participants reported that wearing CH reduced their positive self-image and affected their
417 intimate relationships (P2 and P18). One participant described it as a process of grieving, of
418 trying to get used to a new situation. It decreased quality of life according to P1. P2 stated she
419 had received psychological counseling to accept the disease and its visible effects, including
420 CH. In contrast, others downplayed the impact of CH concerning a serious condition such as
421 cancer that underlies the wearing of CH (P1, P4, P8, P10, P11, and P12).
422 P2: *And what I also find very difficult, I am still single and you still want to meet*
423 *someone special and you don’t feel sexy in such a CH.*
424 P7: *Yes, it’s annoying. It doesn’t combine so nicely. You occasionally see people*
425 *looking at it like: “What is she wearing?” [...] Last Friday I had dinner and I arrived*
426 *by bike in a denim dress with pink CH underneath and they said: “Oh, you look nice*
427 *today, those pink stockings are hip.” Then I said yes they are compression stockings.*
428 *“Oh really?” […] I am not ashamed.*
429 P2: *All the skin-colored ones I do find a pity, […] it still does not match well with your*
430 *normal skin. I still think they stand out a lot.*

431

## 432 Donning and doffing CH

433 Learning to cope with difficulties
434 Many participants had additional conditions (Table 1) that limited their mobility and strength
435 and therefore their ability to don and doff CH. Thirteen participants could don or doff CH by
436 themselves, five received help from an informal caregiver for donning and/or doffing, and four
437 received home care. Four participants did not use an APD, six used gloves and/or a silk foot,
438 eight used a foot slip and two caregivers use the Doff N’ Donner. The participants had varying
439 opinions on the ease of donning and doffing CH. While some found it relatively easy (P1, P4,
440 P13, and P16) and less challenging than expected (P7), others reported that donning can be an
441 arduous task (P2, P3, P5, P10, P14, P17, and P19) that even caused physical complaints (P2,
442 and P17). Donning and doffing require dexterity and proper instruction. The participants
443 reported that when their CH was new, it was very tight. Gradually, it became less tight, making
444 it easier to don and doff (P15). The CH type also matters. According to P2, flat-knit CH is
445 easier to don than round-knit CH, and made-to-measure CH is perceived as tighter than ready-
446 to-wear CH. The participants stated that taking one’s time during donning and doffing is
447 essential; it is more difficult when in a hurry (P2). The participants also mentioned that if the
448 activity was not carried out smoothly, it could cause temporary localized pressure in one area,
449 which could be painful (P12 and P15). Wrinkles were smoothed with anti-slip gloves during
450 the day by some participants (P4, P12, P15, and P18). The participants were conflicted about
451 which activity was the most difficult. According to several of them (P1, P2, P3, P4, and P19),
452 doffing required less effort than donning. However, others found doffing more difficult and
453 exhausting (P6, P7, P10, P14, P15, and P17).
454 Some of the participants needed help at first, but after habituation (P3) or recovery
455 (P15), they could perform independently. The method of donning and doffing changed over
456 time. The participants established their routine and learned tricks, which were not always
457 recommended or optimal for their CH (P2, P4, P5, P7, and P15). For example, P4 used talcum
458 powder to make his legs smoother, and P5 opted for shorter CH instead of tights due to
459 difficulty removing them in time to use the bathroom. Some participants chose less snug (older)
460 CH if they needed to doff and don them multiple times a day, as the effort involved in donning
461 tighter CH was deemed too burdensome. Two participants (P1, P10) reported that physical
462 conditions and social circumstances, such as a surgery or the death of an informal caregiver,
463 may result in temporary or permanent changes that rendered donning and doffing infeasible.
464 The participants indicated that donning and doffing CH affected their self-care routine,
465 requiring more time and prompting reevaluation of morning rituals, such as showering times.
466 Over time, new routines became habitual (P13).
467 P17: *But that donning and doffing… It’s just a disaster. You should see me here alone*
468 *in the evening. And, then I have to put myself in all kinds of twists and turns. I pulled a*
469 *muscle in my but and I can’t do anything.*

470

471 Finding the right place in different circumstances
472 The participants experienced that for donning and doffing, it was important to have a good,
473 stable sitting position, enough space, and a table or cabinet to put all the necessary items. Most
474 people donned and doffed their CH in a fixed place, often in the bedroom or bathroom, sitting
475 on the toilet. The participants mentioned that it was very difficult to don CH under certain
476 circumstances, such as after showering or swimming or donning a tight in a portable toilet at a
477 festival (P2, P4, and P7).
478 I: *Does it matter to you where you don the CH at home?* P2: *I like to be able to sit, so I*
479 *either sit on the toilet or the couch. That way I have space to stretch my legs properly*
480 *and then I sit a little lower so I can bend a little easier. I can also do it standing up, but*
481 *then it takes more strength, sitting down is more comfortable.*

482

483 Appreciating independence
484 The participants noted that when they were fitted for CH, some attention was paid to donning
485 and doffing. However, this attention was often brief and more focused on donning than doffing.
486 If independent donning and doffing was possible, then there was a narrow focus on
487 optimization (P2–P5, P7, P9–P11, P13, and P15). In several cases, there was no attention given
488 at all to donning and doffing or the use of APD (P1, P6, P12, P14, P17, and P18). None of the
489 participants contacted the CT afterwards if donning or doffing was still difficult or became
490 difficult over time. Some participants were unaware that they could seek support and training
491 from an occupational therapist (OT) to learn how to don and doff CH independently (P12, P17,
492 and P19). An OT was only occasionally involved in the home care service to achieve
493 independence (P5 and P9). Sometimes an OT was involved but the participant did not know
494 that this carer was an OT (P9).
495 Independence was important for most participants (P1, P2, P6, P8, P11, P12, P13, P14,
496 P15, P17, and P19). The participants indicated that being dependent on home care means losing
497 your freedom (P6, P9, and P10): Waiting on home care limited their ability to plan the day and
498 to participate in activities outside the home. P2 would even consider surgery if independent
499 donning and doffing was no longer possible. When a partner assisted with donning, the
500 participants and their partners became attuned to each other. Sometimes they did it together
501 (P13). Partners did not see it as a burden (P9, P12, P13). However, not all participants’ partners
502 could provide informal care due to their own health issues or practical limitations (P9).
503 Three participants received home care for donning and doffing CH (P8–P10), and one
504 participant received home care only when her husband had a morning shift (P12). Some
505 participants had already received home care before they received CH because of leg bandages
506 or for assistance with other care activities such as wound care or showering. Sometimes,
507 temporary home care was used (P10) to don and doff CH when the participant was less
508 functional for a while, such as after a fall or surgery. The participants felt that home care
509 workers did not always encourage their clients or informal carers to don and doff CH
510 themselves. Some participants experienced professionals who told them it was not possible to
511 do it independently and took over the care (P8 and P9). According to P5, P9, and P10, home
512 care workers sometimes practiced the use of APD with them to promote self-sufficiency. None
513 of the participants recalled that the full range of APD was presented to them even if self-
514 reliance was not achieved. According to the participants, most home care workers used a foot
515 slip or a Doff N’ Donner to relieve their workload. The participants believed that not all home
516 care workers were skilled at using an APD. In a few cases, the family carer had to explain to
517 the home care worker how to use the APD (P12). According to P8, some home care workers
518 had a personal preference for a particular APD and then took it from client to client. Home care
519 was also provided during holidays, and even abroad (P9).
520 I: *Do you wear the CH the whole day?* P5: *Yeah, from about 7 a.m.* I: *Right when you*
521 *get up?* P5: *No, not right when I get up because I’m already up at 5:30 and then there*
522 *is no help available. So between 7 and 8 in the morning 1 receive help to don the CH.*
523 *I usually wear it until 9 in the evening.*
524 P10: *Yes, of course I want to do that myself because that’s my independence. I mean, I*
525 *want to stay independent for as long as possible.*
526 P9: *The women [home care] who don them also said: “It’s a hopeless task, you can*
527 *hardly get them on yourself.”*
528 P17: *There was a time they [home care] came to don. And then I said: […] “I can’t sit*
529 *with my legs up until 11:30. I’m alone, I have to prepare my food. Because of my*
530 *diabetes, I have to eat regularly.” […] Well, that [waiting on home care] doesn’t work*
531 *for me.*
532 P14: *Yeah, I can do that, but it’s a killer job. I do early shifts and I start at 4:30 in the*
533 *morning. So I get up at 3:30 in the morning and if I want to don the stockings I have to*
534 *get up at least 45 minutes earlier to do this. No home care comes in to help at that time.*
535 *So, I have to don the CH myself. I have these nice rubber gloves that allow me to don*
536 *them properly.*

537

538 Willingness to use an APD depends on the need and perceived benefits
539 Many participants had a positive view of APD and were open to using one if it were effective
540 (P4, P7, P8, P9, P10, and P17); with the exception of P7, they were using an APD at that time.
541 Some participants stated that they would only start using an APD if donning and doffing were
542 to become very difficult or impossible (P1, P2, P6, P7, and P10); they felt they were more
543 restricted when using an APD. The mentioned several reasons for using an APD, including
544 maintaining independence (P10, P13 P15, and P17) and making donning and doffing easier
545 (P4 and P16). The participants indicated that an APD should provide a clear benefit and be
546 easy to use (P2, P7, and P9) and it should not cause frustration (P5 and P9). If the use of an
547 APD caused pain, one participant preferred to accept help (P9).
548 Most participants who had an APD said they used it every day (P4, P12, P13, and P15).
549 APD were used because they made donning and doffing CH quicker and easier (P3, P4, P11,
550 P12, and P16), and to prevent damage to the hand (calluses/blisters) (P2). A few participants
551 did not use their APD daily but chose to when it added value—for example, after showering
552 when legs were still clammy and donning was more difficult (P3). The reasons for not using
553 an APD included no need because donning and doffing could be done without an APD (P1,
554 P2, P7, and P11), using an APD caused stress (P5), being unaware which APD existed (P17
555 and P19), the price of APD (P10), and independent donning/doffing was not possible with the
556 use of an APD (P5, P8 P9, P10).
557 P2: *Yeah, if it can save my back a little bit I would definitely like to try it.* I: *Would you*
558 *also go down the route of getting advice on it?* P2: *Yeah, I think so. If it would help me*
559 *in the future.*
560 P17: *But I’m a little annoyed. That I suddenly hear, thanks to you, that there are people*
561 *who can help you. Who could have the tip of the century? And that all these years, I’m*
562 *71, I’ve had these shitty things in my closet since I was 12, nobody ever told me that.*
563 *That’s a shame.*

564

565 Being unaware of the range of APD
566 According to several participants (P3, P4, P13, P15, and P16), they were provided with a foot
567 slip without consultation, although they did not expect a consultation. In general, the
568 participants were aware of the existence of APD but were not familiar with the whole range of
569 products. Foot slips and frames were mentioned. Non-slip gloves were not recognized as an
570 APD (P1), and arm extending and electric APD were unknown. P17 was completely unaware
571 of the existence of APD. The participants said that in general, they received little information
572 about APD from CTs or home care workers, nor did they look for information themselves.
573 Sometimes, the participants tried several APD before making a choice (P5, P9, and P10).
574 Once the interviewer gave information about the types of APD, several participants
575 expressed great interest and asked for more information (P2, P6, P7, P14, P17, and P19).
576 Several participants intended to contact their CT or an OT as a result of this information. P17
577 emailed a few weeks after the interview to report that she had been seen by an OT and was
578 now able to don and doff her CH independently using Handylegs and a foot slip. As a result,
579 she said show now wears her CH every day instead of occasionally. The participants were often
580 unaware of the cost of an APD and how it is reimbursed (P4, P6, P11, and P15). A few ordered
581 and paid for an APD themselves (P2, P6, and P10), even though it is covered by health
582 insurance. P10 had heard about a new electric APD but did not want to request it because of
583 the cost.
584 P4: *Yeah, I don’t know, are there other APD? Yes, I am very interested. Are they better*
585 *than this one, easier to use than this one?*
586 P10: *They have a new APD now. I heard about it from [name of home care*
587 *organization]. It’s an electric APD to doff the CH, but it’s very expensive. Then I have*
588 *to go to an occupational therapist. I’m thinking, “I’m already so expensive for the*
589 *health insurance company….!”* I: *Well, what do you think home care costs?* P10:
590 *Really?* I: *That’s more expensive in the end than the electric APD. Home care costs*
591 *money every day.* P10: *Every day? Every day?!* I: *Yes, in the end, the electric APD or*
592 *whatever device you use is much cheaper than home care every day.* P10: *I didn’t*
593 *realize that.*

594

595 Satisfied with the use of APD
596 Half of the participants used an APD, mostly a foot slip (for details, see Table 1). The “silk
597 sock” that comes standard with a CH with an open toe was considered less effective than a foot
598 slip by P4, while P1 and P11 preferred this silk sock to a foot slip. The Doff N’ Donner was
599 used by the (informal) carer and not by the participant (P8 and P12).
600 The participants were generally satisfied with the functioning of the APD, mostly a foot
601 slip (P3, P4, P9, P12, P13, and P15). However, some participants had difficulty pulling the foot
602 slip out of the open toe when donning (P4 and P15). The participants did not know of tricks to
603 ease this difficulty. Opinions differed on the durability of a foot slip. P15 was satisfied with the
604 durability whereas P6 and P13 found that it wore out quickly and last only 1 year. P12 thought
605 it is important that an APD fits into a suitcase so that she could take it on holiday.
606 According to P12 and P15, most APD require practice to become proficient, but not all
607 of the participants held the opinion that they had received good instruction (P3, P12, P13, P14,
608 P15, and P17). It was only used once or a few times under supervision during CH collection.
609 After that, the participants had to be able to do it on their own. For P6, the APD was delivered
610 by post without instruction. P12 received the APD from home care, allowing her informal carer
611 to take over, making home care largely unnecessary. In some cases, the use of an APD was not
612 successful at all or caused stress and independence was not feasible (P5 and P9). The
613 participants did not mention anything about evaluation of APD.
614 P5: *I’ve had several APD. One thing you had to slide it over and then you rolled it back*
615 *up and then you rolled it again. That was so many actions and with that. I didn’t get*
616 *that. [...] There was another water thing, a kind of water bag with it. I couldn’t get it to*
617 *go from the head (brain) to the hands. I thought, “forget it.” [...] I also tried a metal*
618 *frame, but then I had to slide the CH over it, but I couldn’t. I didn’t have the strength.*
619 P15: *At the beginning, everything was much more difficult and painful. Because the leg*
620 *was still very swollen and I couldn’t bend the leg properly […]. But I also lost a few*
621 *pounds, so I think that played a part as well. I was also stiffer, I don’t know. And the*
622 *dexterity and the instructions that I’ve gotten since then.*

623

# 624 Discussion

625 In this study, we aimed to delineate the experiences and needs of CH users related to wearing,
626 donning, and doffing CH, and the provision process of CH and associated APD. We identified
627 several subthemes that capture the experiences of becoming and being a CH wearer (or not),
628 wearing CH, and donning and doffing CH. This study has revealed the variance of reactions to
629 wearing CH: The prescription of CH can overwhelm people if there is an acute problem or give
630 them hope if they have been experiencing symptoms for a long time. These differences in
631 responses have not been reported in other studies, which might be a result of a focus on target
632 groups with a single disease in other studies. Based on the findings, becoming a CH wearer has
633 a marked impact because of the negative image of CH (ugly and associated with older age).
634 Finlayson [19] reported in her study that wearers of CH felt associated with disabled people.
635 The finding that CH wearers often feel poorly informed about wearing and maintaining CH has
636 been reported previously [4, 10, 20, 21]. However, this study has added insight into the
637 consequences: For many CH wearers, feeling that they were not well informed caused feelings
638 of insecurity and could result in non-adherence. This is in line with the findings of Finlayson
639 [19]. Although this appears to be obvious and the situation could be easily improved, it is
640 remarkable that the provision of information is still perceived as inadequate.
641 Most of the participants viewed being a CH wearer to be challenging. Issues with
642 discomfort and the appearance of CH causing non-adherence have been presented in other
643 studies [6, 8–10]. In addition to previous studies, we collected the positive and negative effects
644 of wearing CH and explored the impact of these effects on daily life and psychological well-
645 being. For example, the tendency of CH to sag is well known, but the effect of having to lift
646 CH when away from home has not been described previously. This perspective broadens our
647 understanding of the reasons users choose not to wear CH and provide points to address to
648 ensure clients are better informed.
649 We found several ways users dealt with the discomfort of wearing CH, including wearing CH
650 only if edema increased or as a preventive measure when edema is expected to increase due to
651 weather or activity. These strategies have also been reported by Gong et al. [6] and Probst et
652 al. [10].
653 Despite the availability of APD, the participants in this study shared problems with
654 donning and doffing CH, a finding similar to previous studies [7, 10, 20, 21, 22]. Some
655 participants accepted help from informal carers or home care because they felt there was no
656 alternative. However, not everyone accepts the loss of independence and, therefore, they may
657 decide not to wear CH, even though not wearing CH has physical risks. Probst et al. [10] and
658 Chitambira [21] also described that the acceptance of professional support and the resulting
659 loss of autonomy can be a barrier to wearing CH. We found that our participants were not well
660 informed regarding many APD; they reported not being informed about APD during the CH
661 fitting process. Previous studies have also shown that CTs and OTs are not fully aware of the
662 range of APD and their usability [23]. The lack of knowledge about APD may be one of the
663 reasons why the potential of APD is not fully exploited and only a relatively small proportion
664 (15%) of those who wear CH use an APD [3]. More attention to the range of APD and their
665 applicability in the vocational training and continuing education of CTs, OTs, and district
666 nurses may have a positive impact on the independence of CH wearers.
667 Due to its classification as a medical device, individuals in need of CH transition from
668 being consumers with free choice to patients with limited choice: They need care and
669 reimbursement from health insurance companies [24]. Some participants in this study
670 described the process of becoming a CH wearer as one of grieving and trying to get used to a
671 new situation. In contrast, others downplayed the impact of CH regarding their serious
672 condition. Our findings indicate that many factors impact the acceptance of CH. Next to the
673 physical effect of wearing CH, the appearance of CH and the effect on self-image frequently
674 plays a major role. Vreeswijk [24] noted that the relationship between the appearance of the
675 assistive product, the cultural context, and current fashion influence the meaning users attribute
676 to an assistive product. CH are visible and part of clothing, but they can hardly be adapted to
677 one’s fashion preferences. Just as Vreeswijk [24] described, our participants stated that they
678 felt unattractive, embarrassed, and old wearing CH, and they placed value on the choice of
679 color of the CH. Vreeswijk [24] explained that the appearance of CH is important in coping
680 with a new situation and disciplining oneself to wear CH every day. So, the CH color choice is
681 not simply a “luxury”; rather, it is a factor that influences effectiveness because it plays a role
682 in acceptance and participation in daily life situations [24].
683 Most participants said they wear CH consistently due to the benefits they experience.
684 For some, motivation lay in the fear of the consequences of not wearing CH, such as wounds
685 or recurrent thrombosis or varicose veins. These findings are consistent with previous studies
686 [6, 10, 21]. The participants who did not comply stated the following reasons for their non-
687 adherence: lack of short-term benefits, appearance, comfort, and inability to don and doff CH
688 independently. Finlayson [19] reported high rates of non-adherence (47%) largely explained
689 by difficulties with donning or doffing, misconceptions about function and wearing duration,
690 and problems related to the unaesthetic appearance of CH. The participants expressed their
691 willingness to use an APD if it were to provide a clear benefit. If not, then the convenience of
692 using an APD does not outweigh the impact of using it, such as feeling more restricted and
693 experiencing stress. The listed factors for accepting the use of both CH and APD correspond
694 to the determinants—perceived usefulness, ease of use, and benefits—that have been identified
695 as success factors for the adoption of a product in several specific models such as the
696 technology acceptance model [25] and the unified theory of acceptance and use of technology
697 [26]. Other models like the diffusion of innovation model [27] and the model of pre-
698 implementation acceptance [28] also emphasize the influence of the social environment on the
699 decision to use a product. Our study also revealed how the social environment impacted the
700 use of CH and APD.
701 Based on our findings, more attention should be paid to the many factors that contribute
702 to the process of becoming a CH wearer. To increase adherence, caregivers should not only
703 support the use of products, but also focus on acceptance of the new self-image. Mayer-Larssen
704 et al. [15] recommended a client-centered approach in the provision of assistive products. The
705 provision process might benefit from the model of Bloem and Stalpers [29], which emphasizes
706 the relevance of assessing a client’s motivation and readiness and collaboratively setting goals
707 and action plans.
708 The participants remembered having received a lot of verbal information during the CH fitting
709 appointment, but most were unable to recall exactly what was said. Some participants held
710 incorrect beliefs such as tight trousers enhance the effectiveness of CH, machine washing and
711 spinning should be avoided, and CH color influences its effectiveness. The information leaflet
712 attached to the CH did not meet the needs of the CH wearers. Berszakiewicz et al. [4], Probst
713 et al. [10], and Perry et al. [20] have endorsed the importance of educational interventions with
714 a focus on patient perceptions and understanding. Berszakiewicz et al. [4] and Dale and Gibson
715 [30] have even related non-adherence to a lack of understanding of how CH works. We found
716 that participants have different needs regarding information about CH and APD, and that
717 adherence to wearing CH is about changing behavior, acceptance, and dealing with negative
718 effects of CH. According to Bloem and Stalpers [29], changing habits requires coaching;
719 moreover, the delivery of information should not be standard, but rather based on the
720 acceptance of the chronic illness and the client’s perceived control.
721 Based on our study, some alterations in the provision process of CH and APD might be
722 beneficial to increase adherence to wearing CH and independence in donning and doffing.
723 Users might benefit from objective, non-commercial, easy-to-read information about the
724 functioning of CH, donning and doffing CH, and maintenance. Both digital and non-digital
725 information are necessary, as many CH wearers are elderly and not digitally proficient. More
726 emphasis is needed on the acceptance and impact of wearing CH. Although not fully aware of
727 the range of possibilities, the participants were very interested in APD to maintain their
728 independence. A previous study identified a knowledge gap among healthcare professionals
729 about APD and bottlenecks in the reimbursement structure of CH and APD [23]. CT education
730 could be extended to include the psychological aspects of wearing CH, as well as training about
731 APD. However, adequate reimbursement of the time spent for educating and training CH
732 wearers and adequate reimbursement of APD is necessary to motivate CTs to apply the new
733 strategies in the provisioning process. As healthcare costs continue to rise and staffing becomes
734 problematic, it would be very useful to study whether changes in the provision process could
735 increase adherence to CH and independence in donning and doffing.

736

## 737 Strengths and limitations

738 In contrast to most other studies, we centered our research on people who wear CH regardless
739 of the underlying medical condition. A benefit of the diversity in medical conditions is that we
740 collected wide of experiences wearing CH and the impact of CH. A major strength is that we
741 also included participants who did not (fully) adhere to wearing CH. These interviews provided
742 interesting information, particularly about the reasons for non-adherence. The disadvantage
743 was that the non-adherent participants had often been prescribed CH more than 3 years ago,
744 which may have affected their memory of the provision process, possibly causing response
745 bias.
746 Due to the wide variation in gender, condition, type of CH, and adherence, we captured
747 a wide range of experiences. Admittedly, the sample is not representative of the general Dutch
748 population that uses CH. The average age and the representation of people with venous disease
749 were lower in our sample compared with the overall population. Further, we only interviewed
750 white native Dutch people. Although we tried, we were unsuccessful in including participants
751 with other ethnic backgrounds in the study. Evaluating the representativeness of the
752 participants proved to be challenging due to the absence of dependable Dutch data regarding
753 the occurrence of CH across various ethnicities.
754 The fact that the interviewer is a CH wearer herself and thereby an expert by experience
755 brought challenges in addition to the benefits already described, such as being able to
756 empathize and recognize experiences and thus ask probing questions to get deeper insights.
757 Particular vigilance was required to remain objective and not to ask suggestive questions.
758 Nevertheless, the advantages of this situation outweighed the disadvantages. Personal
759 reflexivity was applied throughout the entire study by explicitly disclosing the researcher’s
760 prior experiences and motivations [31].
761 The interviews were conducted in 2022 shortly after the coronavirus disease 2019
762 (COVID-19) pandemic restrictions in the Netherlands had been lifted. Some participants
763 received a CH for the first time during this pandemic. Due to the restrictive measures, the
764 provisioning process may have been different from normal. For example, in some cases the
765 partner was not allowed to be present during appointments with the physician or CH fitting.

766

# 767 Conclusion

768 We found that wearing CH has a large impact on a person’s life due to comfort, appearance,
769 and possible loss of independence. Poor comfort—such as sagging, stretch marks, and heat—
770 is a major reason for not wearing CH. The effect of the appearance of CH on self-image is an
771 important but underestimated factor regarding the acceptance of CH. The participants reported
772 feeling unattractive, embarrassed, and old wearing CH. CH are visible and part of clothing, but
773 can hardly be adapted to one’s fashion preferences. The participants placed value on the CH
774 color choice. To increase adherence, we recommend to take this into account when determining
775 the number and design of CH eligible for reimbursement.
776 The reason why wearing CH is important is not always clear, and CH wearers often feel
777 not well informed about wearing and washing instructions. There is a need for independent
778 information. It should be considered that participants have different needs regarding
779 information based on their acceptance of the chronic illness and desired level of self-
780 management, so information and guidance should be tailored.
781 Independence in donning and doffing CH is important for clients, and loss of
782 independence is a major reason for non-adherence. The use of APD can prevent dependence,
783 but, in general, the full range of APD have not been exploited. More attention to donning and
784 doffing CH and APD during the fitting appointment of CH is recommended. In addition, there
785 should be easy-to-find and accessible objective, non-commercial information on the
786 possibilities of APD.

787

# 788 Acknowledgements

789 We would like to acknowledge several people:
790 •	All of the participants for candidly sharing their experiences;
791 •	Lymfoedeem.nl, Harteraad, Ron van Bekkum, Suze van Beusekom, and Robert
792 Meinders for their help in recruiting participants;

| 793 | - Tessa, Iris, and Kirsten for transcribing the interviews; and |
| --- | --- |
| 794 | - ZonMw for the grant that made this research possible. |
| 795 |  |
| 796 | **References** |
| 797 | 1. Clark M, Krimmel G. Template for practice: Compression hosiery in lymphoedema. |
| 798 | London: MEP Ltd; 2006. |
| 799 | 2. Rabe E, Partsch H, Hafner J, Lattimer C, Mosti G, Neumann. M, Urbanek T, Huebner M, |
| 800 | Gaillard S, Carpentier P. Indications for medical compression stockings in venous and |
| 801 | lymphatic disorders: an evidence-based consensus statement. Phlebology. 2018;33(3): |
| 802 | 163–184. doi:10.1177/0268355516689631 |
| 803 | 3. Zorginstituut Nederland. Gipdatabank. [Internet]. [cited 24 May 2024]. Available: |
| 804 | [https://www.gipdatabank.nl](https://www.gipdatabank.nl/) |
| 805 | 4. Berszakiewicz A, Kasperczyk J, Sieroń A, Krasiński Z, Cholewka A, Stanek A. The effect |
| 806 | of compression therapy on quality of life in patients with chronic venous disease: |
| 807 | a comparative 6-month study. Postepy Dermatol Alergol. 2021;38(3): 389–395. |
| 808 | doi:10.5114/ada.2020.92277 |
| 809 | 5. Kankam H, Lim C, Florentino F, Davies A, Gohel M. A Summation analysis of compliance |
| 810 | and complications of compression hosiery for patients with chronic venous disease or post- |
| 811 | thrombotic syndrome. Eur J Vasc Endovasc Surg. 2018;55: 406–416. |
| 812 | 6. Gong JM, Du JS, Han DM, Wang WY, Qi SL. Reasons for patient non-compliance with |
| 813 | compression stockings as a treatment for varicose veins in the lower limbs: A qualitative |
| 814 | study. PLoS One. 2020;15(4): e0231218. doi:10.1371/journal.pone.0231218 |
| 815 | 7. Sippel K, Seifert B, Hafner J. Donning devices (foot slips and frames) enable elderly people |
| 816 | with severe chronic venous insufficiency to put on compression stockings. Eur J Vasc |
| 817 | Endovasc Surg. 2015;9: 221–229 |

| 818 8. | Van Hecke A, Grypdonck M, Defloor T. A review of why patients with leg ulcers do not |
| --- | --- |
| 819 | adhere to treatment. J Clin Nurs. 2009;18: 337–349 |
| 820 9. | Mofatt C, Dorman M. Recurrence of leg ulcers within a community ulcer service. J Wound |
| 821 | Care. 1995;4(2): 57–61. |
| 822 10. | Probst S, Séchaud L, Bobbink P, Buehrer-Skinner M, Weller C. The lived experience of |
| 823 | recurrence prevention in patients with venous leg ulcers: An interpretative |

824 phenomenological	study.	J	Tissue	Viability.	2021;29(3):	176–179.
825 doi:10.1016/j.jtv.2020.01.001
826 11. Savin-Baden M, Howell Major C. Qualitative research – The essential guide to theory and
827 practice. London: Routledge; 2013.
828 12. Frambach J, Van der Vleuten C, Durning S. Quality criteria in qualitative and quantitative

829	research. Acad Med. 2013;88(4): 552

830	13. Korstjens I, Moser B. Series: Practical guidance to qualitative research. Part 2: Context,

831	research	questions	and	designs.	Eur	J	Gen	Pract.	2017;23(1):	274–279.

| 832 | doi:10.1080/13814788.2017.1375090 |
| --- | --- |
| 833 14. | Nictiz. Procesbeschrijving Hulpmiddelenzorg. Den Haag: Nictiz; 2009. |
| 834 15. | Mayer-Larsen S, Falgreen-Mortensen R, Kaae-Kristensen H, Hounsgaard L. Older adults’ |
| 835 | perspectives on the process of becoming users of assistive technology: A qualitative |
| 836 | systematic review and meta-synthesis. Disabil Rehabil Assist Technol. 2019;14(2): 182– |
| 837 | 199. |
| 838 16. | Hsieh H, Shannon S. Three approaches to qualitative content analysis. Qual Health Res. |
| 839 | 2005;15(9): 1277–1288. doi:10.1177/1049732305276687 |
| 840 17. | Mayring P. Qualitative content analysis. Forum Qual Soc Res. 2000;1(2). |

841	18. Moser A, Korstjens I. Series: Practical guidance to qualitative research. Part 3: sampling,

842	data	collection	and	analysis.	Eur	J	Gen	Pract.	2018;24(1):	9–18.

| 843 | doi:10.1080/13814788.2017.1375091 |
| --- | --- |
| 844 19. | Finlayson K, Edward H, Courtney M. The impact op psychosocial factors on adherence to |
| 845 | compression therapy to prevent recurrence of venous leg ulcers. J Clin Nurs. 2010 |
| 846 | May;19(9-10):1289-97. doi:10.1111/j.1365-2702.2009.03151.x |
| 847 20. | Perry C, Atkinson RA, Griffiths J, Wilson PM, Lavallée JF, Cullum N, Dumville JC. |
| 848 | Barriers and facilitators to use of compression therapy by people with venous leg ulcers: A |
| 849 | qualitative exploration. J Adv Nurs. 2023;79: 2568–2584. doi:10.1111/jan.15608 |
| 850 21. | Chitambira F. Patient perspectives: Explaining low rates of compliance to compression |
| 851 | therapy. Wound Pract Res. 2019;27(4): 168–174. doi:10.33235/wpr.27.4.168-174 |
| 852 22. | Hampton S. Acti-Glide: A simple method of applying compression hosiery. Br J |
| 853 | Community Nurs. 2004;10(5): 244–246. |
| 854 23. | Hagedoren E, Roentgen U, Van der Heide L, Zwakhalen S, Daniels R. Challenges in the |
| 855 | provision process of assistive products for donning/doffing compression stockings as |
| 856 | perceived by all stakeholders. In Petz A, Hoogerwerf EJ, Mavrou K, editors. ICCHP- |
| 857  858 | AAATE 2022 open access compendium “Assistive Technology, Accessibility and  (e)Inclusion” Part II. Linz: JKU Universitätsbibliothek; 2022. pp. 53–59. |
| 859 | doi:10.35011/icchp-aaate22-p2-07 |
| 860 24. | Vreeswijk P. Lichaam en hulpmiddel. Amsterdam: Edmund Husserl-Stichting; 2002. |
| 861 25. | Davis F. Perceived usefulness, perceived ease of use, and user acceptance of |
| 862 | information technology. MIS Q. 1989;13(3): 319–340. |

863 26. Venkatesh V, Morris M, Davis F, Davis D. User acceptance of information technology:
864 Toward a unified view. MIS Q. 2003;27(3): 425–478.
865 27. Rogers E. Diffusion of innovations. New York: Free Press; 1995
866 28. Peek D, Wouters E, Hoof J, Luijkx K, Boeije H, Vrijhoef N. Factors influencing acceptance
867 of technology for aging in place: A systematic review. Int J Med Inform. 2014;83: 235–
868 241.
869 29. Bloem JG, Stalpers JFG. Subjective experienced health as a driver of healthcare behaviour.
870 Nyenrode Research Papers Series. 2012;12(01): 1–22. doi:10.2139/ssrn.2102513
871 30. Dale J, Gibson B. Information will enhance compliance; informing clients about

872	compression hosiery. Prof Nurse.1992;7(11): 755–756,758–760

873	31. Olmos-Vega FM, Stalmeijer RE, Varpio L, Kahlke R. A practical guide to reflexivity in

874	qualitative research: AMEE Guide No. 149. Med Teach. 2023;45(3): 241–251.

875	doi:10.1080/0142159X.2022.2057287
