# Supplementary material for: The impact of wearing compression hosiery and the use of assistive products for donning and doffing: A descriptive qualitative study into user experiences
Source: PLoS One. 2024 Dec 26;19(12):e0316034. doi: 10.1371/journal.pone.0316034 (PMC11670924; doi:10.1371/journal.pone.0316034)
Supplement: Attachment — Submitted filename: recommandations.docx [file pone.0316034.s003.docx]

Very good article

The structure of the article looks like a market survey.

Recommendations for manufacturers and public and private insurers are lacking at the end of the article
