# Supplementary material for: The impact of wearing compression hosiery and the use of assistive products for donning and doffing: A descriptive qualitative study into user experiences
Source: PLoS One. 2024 Dec 26;19(12):e0316034. doi: 10.1371/journal.pone.0316034 (PMC11670924; doi:10.1371/journal.pone.0316034)
Supplement: Attachment — Submitted filename: Response to Reviewers .docx [file pone.0316034.s004.docx]

**Rebuttal letter to the editor and reviewers**

We would like to express our gratitude for your valuable feedback on our manuscript. It is encouraging to hear that it has been rated as good and useful by Reviewer 2.

Your recommendations help us to improve the manuscript and we changed it accordingly. The following section will address the manner in which the feedback provided by the academic editor and reviewers has been incorporated into the text.

The style requirements were subjected to a process of verification and subsequent modification where necessary.

The compression classes were specified with measure of compression in mmHg.

In response to the request from Reviewer 1, an image illustrating the various types of APD was incorporated into the text.

The frameworks used to identify the topics in the interview guide were explained in text and illustrated with figures. The figures were verified with the PACE system to guarantee adherence to the PLOS standards. We asked permission to re-use the figures taken from other publications, but one permissions is not granted, yet. We have the full rights for the illustrations in figure 1. We received permission from Nictiz to re-use figure 2 and are waiting for the permission of Taylor & Francis Journal Permissions to re-use figure 3. We will inform you as soon as we have received it from the publisher.

The reference list was subjected to a review, during which any necessary modifications were implemented. Our search did not identify any papers that had been retracted.

Availability of the data: We have sought the approval of the Medical Ethical Review Committee (METC Z) and the Data Protection Officer of Zuyd University of Applied Science for the sharing of anonymised data in a public forum. The aforementioned bodies have granted permission for this data to be made publicly available, on the condition that it cannot be traced back to individuals. To ensure this, the participants' professions have been removed from the transcripts. Zuyd University will shortly be utilising DataverseNL for this purpose. The DOI link will be provided as soon as DataverseNL is operational.

The interview guide was made available in English as supporting information.

The corrections proposed by Reviewer 1 have been incorporated into the manuscript and are visible in the track changes version. In accordance with the advice provided by Reviewer 1, recommendations for manufacturers and health care insurers have been added to the conclusion section of the manuscript.
